# Effect of different organic fertilizers application on growth and environmental risk of nitrate under a vegetable field

**DOI:** 10.1038/s41598-017-17219-y

**Published:** 2017-12-05

**Authors:** Shuyan Li, Jijin Li, Bangxi Zhang, Danyang Li, Guoxue Li, Yangyang Li

**Affiliations:** 1 Beijing Key Laboratory of Farmland Soil Pollution Prevention and Remediation, College of Resources and Environmental Science, China Agricultural University, Beijing, 100193 China; 2 Institute of Plant Nutrition and Resources, Beijing Academy of Agriculture and Forestry Sciences, Beijing, 100097 China; 3 Guizhou Institute of Soil and Fertilizer, Guiyang, 550006 China

## Abstract

The effect of chicken manure after different disposal methods (water-logged composting, GOF; anaerobic digestion, BR; thermophilic composting, ROF) on vegetable growth and environmental risk was investigated under the tomato-celery-tomato field. Results showed that organic fertilizers significantly increased vegetable yield and quality, but with inappropriate application may cause serious environmental risk such as nitrate pollution. Maximum vegetable yield of 80.9, 68.3, 112.7 t·ha^−1^ (first, second and third rotation crop, respectively) with best vegetable quality was obtained in ROF treatment. The highest N use efficiency with the least nitrate enrichment in soil was also found in ROF treatment. Moreover, under this fertilization way, nitrate concentration in soil leachate dropped to 6.4 mg·L^−1^, which satisfied the threshold (<10 mg·L^−1^) for drinking water set by the US Environmental Protection Agency. Thus, ROF was suggested to be the optimal fertilizer with the best yield, quality and the least environmental risk under the ^“^tomato-celery” rotation system.

## Introduction

Although nitrogen (N) utilization has generally been optimized in agriculture, unreasonable fertilization can lead to agricultural non-point source pollution^1–6^, and improvements are necessary to avoid adverse environmental impacts of nitrate leaching. Nitrate leaching has a significant influence on plant N supply and groundwater quality. Nitrate concentrations in soil depend on the relation between uptake by plants, soil organisms, atmospheric N_2_ fixation, N mineralization (ammonification and nitrification), N deposition from the atmosphere, denitrification, and volatilization^7^.

The development of intensive agricultural areas based on irrigation with groundwater and N application in farming areas has had serious side effects on the land ecosystems including ground water depletion and nitrate leaching to ground water^6,8^. Due to environmental pollution, high nitrate concentrations may accumulate in the edible parts of some vegetables, particularly if excessive N fertilizer has been applied^9^. Consuming these crops can harm human health.

Leaching of nitrate from soil is driven by land-use type, management (e.g., fertilization), land-use change, climate, and soil properties^10^. Nitrate-N leaching losses were usually less from fine-textured soils than from coarse-textured soil^11^. The soil nitrate content may higher in spring than in autumn^12^. Precipitation/irrigation can significantly increase the nitrates in the soil leachate^13,14^. Nitrate losses decreased with the drain depth decreased^15^.

Organic fertilizers have been proposed as one solution to relieve environmental pressure and be a carbon-neutral alternative to liquid fossil fertilizers^16^. Organic matter improves soil structure, increases the water holding capacity and promotes biological transformations such as N-mineralization^16,17^. Several researchers have examined the impact of timing of N and water applications on crop yield in field experiments^8,18^. Behnke *et al*.^19^ found that N annual losses from 22.7 to 59.9 kg·ha^−1^, and they increase with N fertilization rates increase. The soil NO_3_^−^-N content under basal fertilizer was 1.65 times higher than that without fertilizer at 0–10 cm on the 36th day after sowing^20^. Davis *et al*.^21^ found that N applications increased N leaching and N_2_O emission without increasing biomass production. Liu *et al*.^9^ found that lettuce augmented with organic fertilizers had significantly longer and wider leaves, higher shoot, and lower NO_3_^−^-N concentrations compared with the same amount of inorganic fertilizers. Guo *et al*.^22^ found that N fertilizers coupled with farm yard manure resulted in 70% less NO_3_^−^-N accumulation in the soil profiles than that using mineral N fertilizer alone. However, some researches had found that manure applications without any pretreatment could cause serious NO_3_^−^-N leaching^23,24^.

There have been many kinds of organic fertilizers, such as manure, sewage sludge, stalks, compost, biogas residues, biogas slurry and so on. An increasing body of literature has been focused on the N fertilizers for crop yield and NO_3_^−^-N leaching, but very little is about comparing different kinds of organic fertilizers on NO_3_^−^-N distribution (soil, leachate and crop), vegetable yield and quality during the agricultural process. To solve the problem of nitrate content in vegetables, soil and underground water exceeding standard caused by unreasonable fertilization, specific objectives of this study were to: (i) evaluate different organic fertilizers on vegetable yield and quality; (ii) and also determine nitrate concentrations in different soil layers and soil leachate to evaluate environmental risk.

## Results and Discussion

### Vegetable yield

**Figure 1:**
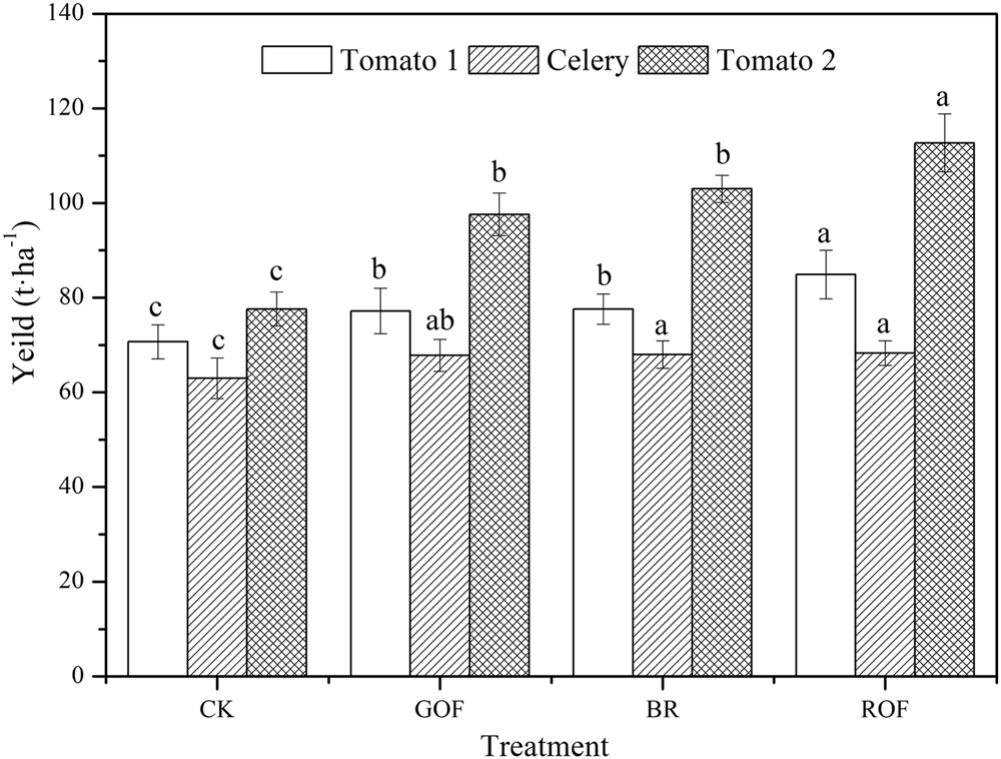
Effect of different organic fertilizers on vegetable yield. Mean differences in the bars are significant at P_0.05_ level with different letters. Tomato 1 means the first rotation vegetable; Celery means the second rotation vegetable; Tomato 2 means the third rotation vegetable. Repeat in following figure.

As expected, organic fertilizers significantly increased vegetable yield by 7.6–45.2% (Fig. 1). For the first rotation, tomato yield increased by 9.2–20.1% compared with CK. Among this, ROF did best with the yield of 84.9 t·ha^−1^ and it was significantly higher than other treatments. However, GOF had the least effect on tomato yield and BR was similar to GOF with the increase of 9.8%. In the second rotation, all the treatments increased the celery yield by 7.6–8.4%. The maximum increase was ROF treatment with the production of 68.3 t·ha^−1^. Compared with tomato, the increase of celery was not obvious. After application of organic fertilizers for one year, the tomato yield in all the treatments in this rotation increased compared with the first rotation. This was mainly due to the higher N mineralization as a result of higher biological activity^25^. For the third rotation crop, tomato yield increased by 25.8–45.2% compared with CK. In this time, ROF has the maximum yield with 112.7 t·ha^−1^. Among this “tomato-celery-tomato” system, organic fertilizers significantly increased the vegetable yield according to ANOVA test. This may because organic fertilizer application can increase soil organic matter and then increase yields^26,27^.

### Vegetable quality

**Figure 2:**
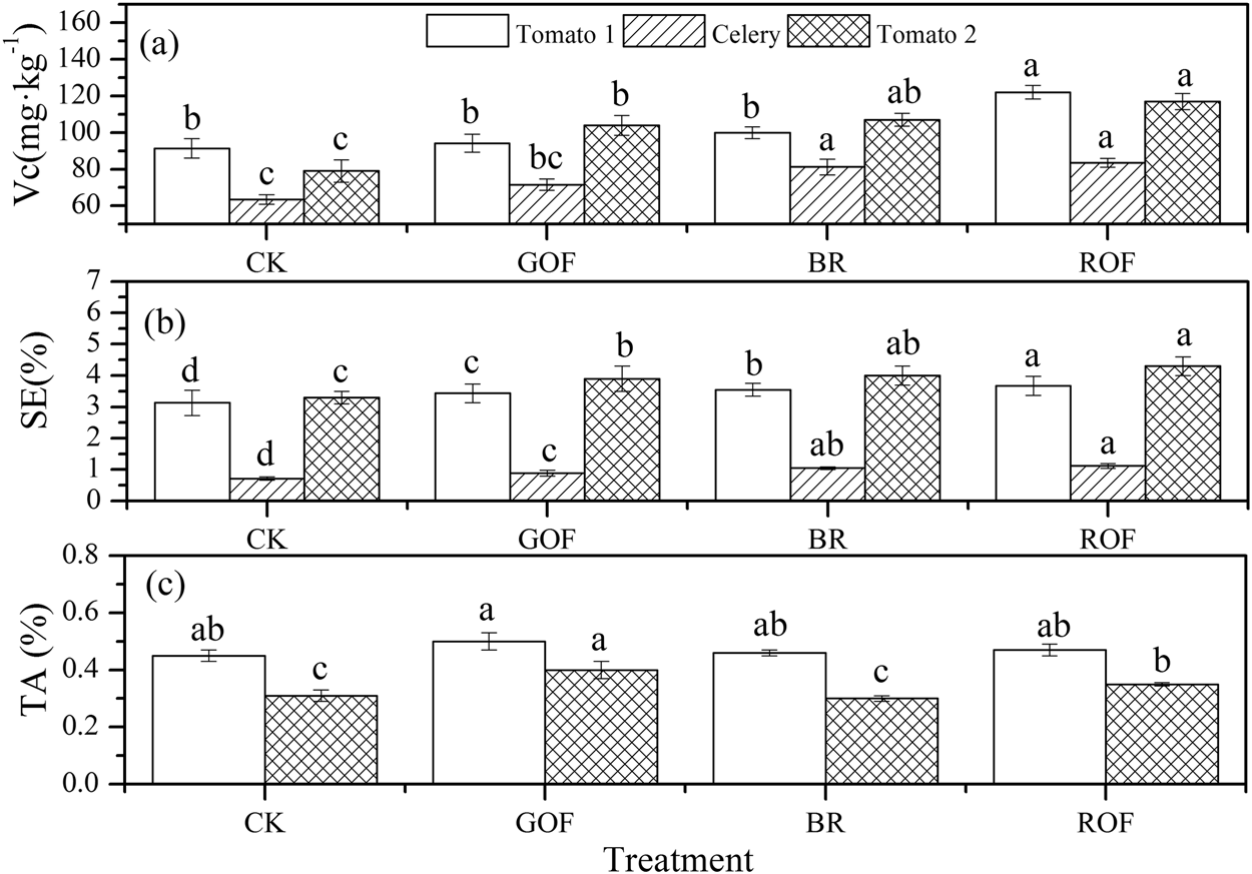
Effect of different organic fertilizers on vegetable quality Vc: vitamin C; SE: soluble sugar; TA: titratable acidity.

Application of organic fertilizers can increase vegetable qualities (Fig. 2). The concentration of vitamin C (Vc) after harvest the vegetables is shown in Fig. 2a. Application organic fertilizers significantly increased the concentration of Vc by 3.0–33.5% in the first rotation crop. ROF with the Vc concentration of 122 mg·kg^−1^ had the best effect and GOF did worst. This may because GOF had a low humification degree without a thermophilic phase^9^. The concentration of Vc increased by 12.6–31.5% in celery planting. Like the first rotation, ROF with the concentration of 83.5 mg·kg^−1^ had the best effect and GOF had the least effect. After three rotations, Vc of tomato in CK treatment decreased from 91.4 (first rotation) to 79.0 (third rotation) mg·kg^−1^, indicating undernourishment and N depletion. Organic fertilizers application could increase the concentration of Vc by 31.6–48.1% compared with CK in the third rotation. ROF with the concentration of 117 mg·kg^−1^ had the best effect. This is mainly because ROF with a high stabilization and humification degree could improve soil structure, increase the water holding capacity and promote biological transformations and then improve the vegetable quality^9^.

Figure 2b gives the concentration of soluble sugar (SE) after harvest the vegetables. Application organic fertilizers can significantly increase the concentration of SE by 9.9–17.3% in the first rotation. ROF had the best effect with the concentration of 3.67%. The concentration of SE was increased by 23.6–55.6% in celery planting. ROF with the concentration of 1.12% had the best effect among all the treatments, and GOF had the least effect of all. In the third rotation, the concentration of SE was increased by 18.2–30.3%. Similar to the celery, ROF with the concentration of 4.30% had the best effect of all, and GOF had the least effect. The concentration of SE with the third rotation increased in all treatments including CK compared with the first rotation. This illustrates that long-term application of organic fertilizer can improve the quality of vegetables.

The concentration of titratable acidity (TA) after harvest tomatoes is shown in Fig. 2c. Organic fertilizers application has no significant influence on TA. The content of TA in the third rotation decreased compared with the first rotation, indicating that organic fertilizer can improve the vegetable taste.

### Nitrate concentration in vegetable

**Figure 3:**
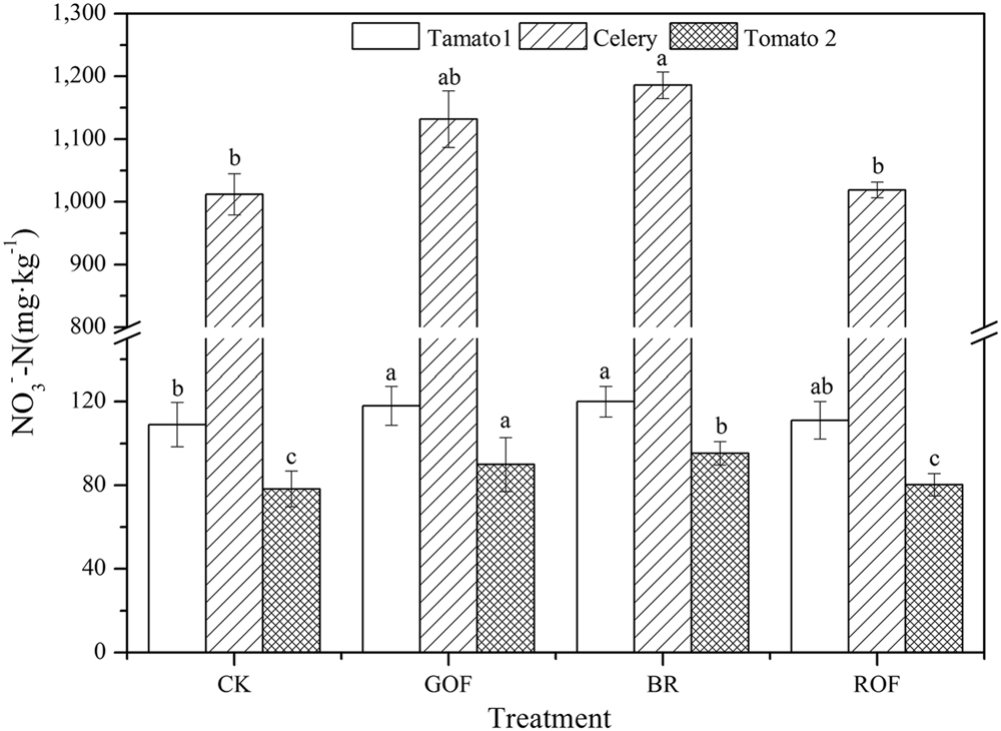
Effect of different organic fertilizers on nitrate content of tomato and celery.

NO_3_^−^-N concentration is an important quality characteristic of vegetable. NO_3_^−^ was perceived as a purely harmful dietary component which causes infantile methaemoglobinaemia, carcinogenesis and possibly even teratogenesis^28^. Figure 3 gives the NO_3_^−^-N concentration in tomato and celery. From this, NO_3_^−^-N concentrations of the two rotations of tomato were all less than 120 mg·kg^−1^ especially in the latter, which were far less than the limit of the national standard 600 mg·kg^−1^ (GB18406.1-2001). Celery is a crop which is easy to enrich NO_3_^−^ and this is why the NO_3_^−^-N concentration in the third rotation of tomato lower than the first rotation especially in CK treatment. NO_3_^−^-N concentration in celery was much higher than that in tomato, but it was still less than the limit of the national standard 3000 mg·kg^−1^. Tomato-celery rotation system could significantly decrease the vegetable NO_3_^−^-N concentration under this continuous fertilization field. Through these three rotations, ROF had no significant difference with CK in terms of vegetable NO_3_^−^-N concentration, which indicates that ROF is a relatively safe way for fertilizing.

### Nitrate content in soil

#### Nitrate concentration in the 0–60 cm soil layers (in time)

**Figure 4:**
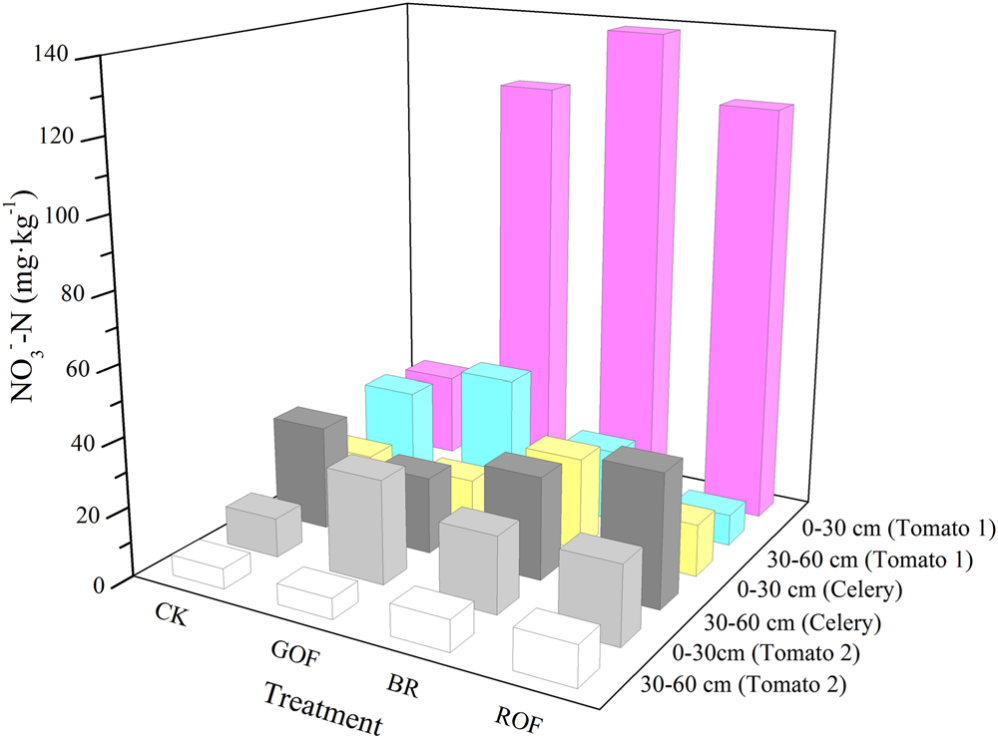
Nitrate content in 0–60 cm soil profile after harvest vegetables (with time).

NO_3_^−^-N concentration in the root zone soil of the three rotations are shown in Fig. 4. Organic fertilizers significantly affect the soil nitrate concentration in the top layers (0–60 cm). The NO_3_^−^-N concentration in the 0–30 cm soil layer of GOF and ROF treatments achieved the minimum value after harvest the second rotation (celery). Celery roots were mainly distributed in 0–30 cm^29^ resulting in less absorbing of NO_3_^−^-N below 30 cm soil layer. This may be the reason why the NO_3_^−^-N concentration in 30–60 cm soil layer higher than that in 0–30 cm soil layer after harvest celery. Furthermore, for the second rotation NO_3_^−^-N content in the top 30 cm soil layer reduced by 60–80% compared with the first rotation, but NO_3_^−^-N in the 30–60 cm soil layer may have a small amount of accumulation for little absorption. However, there were no significant differences in the soil NO_3_^−^-N content in terms of the whole top layers (0–60 cm) compared with CK, due to NO_3_^−^-N absorption and enrichment in celery. The soil NO_3_^−^-N concentration after harvest the first rotation of tomato was significantly higher than the other rotations. This may be caused by the high nitrate content of the original soil (Table 1). Organic fertilizers application significantly increased the NO_3_^−^-N concentration in the 0–30 cm soil layer of all treatments after the first rotation, and especially in BR reached 138.2 g·kg^−1^, indicating a high risk of leaching. However, NO_3_^−^-N in the 30–60 cm soil layer changed slightly due to root absorption. After third rotation, NO_3_^−^-N concentration was significantly lower than the first rotation, owing to the low nitrate background values in this rotation after harvest celery. In this time, NO_3_^−^-N concentration in the 0–30 cm soil layer increased slightly. Moreover, NO_3_^−^-N concentration in the 30–60 cm reduced significantly, which could be attributable to the higher absorption of N in the 30–60 cm soil layer by the deeper root of tomato^30^. Thus in this tomato- celery rotation system, long time application of organic fertilizers will not affect soil nitrate content in the top layers (0–60 cm).

**Table 1:** Characteristics of original soil.

Physicochemical indices	Concentration
Soil texture	Clay loam
Clay (%)^a^	22.8 ± 0.8
Silt (%)^a^	43.2 ± 1.2
Sand (%)^a^	34.0 ± 0.5
pH	8.07 ± 0.07
Organic matter (g·kg^−1^)^a^	19.0 ± 0.2
Total N (g·kg^−1^)^a^	1.34 ± 0.06
Alkali-hydrolyzable N (mg·kg^−1^)^a^	116 ± 4
Olsen-P (mg·kg^−1^)^a^	105 ± 3
Rapidly available K (mg·kg^−1^)^a^	248 ± 8
Available Cu (mg·kg^−1^)^a^	3.35 ± 0.07
Available Zn (mg·kg^−1^)^a^	5.64 ± 0.06
Available Ca (mg·kg^−1^)^a^	3560 ± 42
Available Fe (mg·kg^−1^)^a^	31.4 ± 1.3
Available Mn (mg·kg^−1^)^a^	4.21 ± 0.07
Available Mg(mg·kg^−1^)^a^	2370 ± 35

Values are given as the mean ± standard deviation (n = 3). ^a^Based on dry matter (DM).

#### Nitrate content in soil profile (in depth)

**Figure 5:**
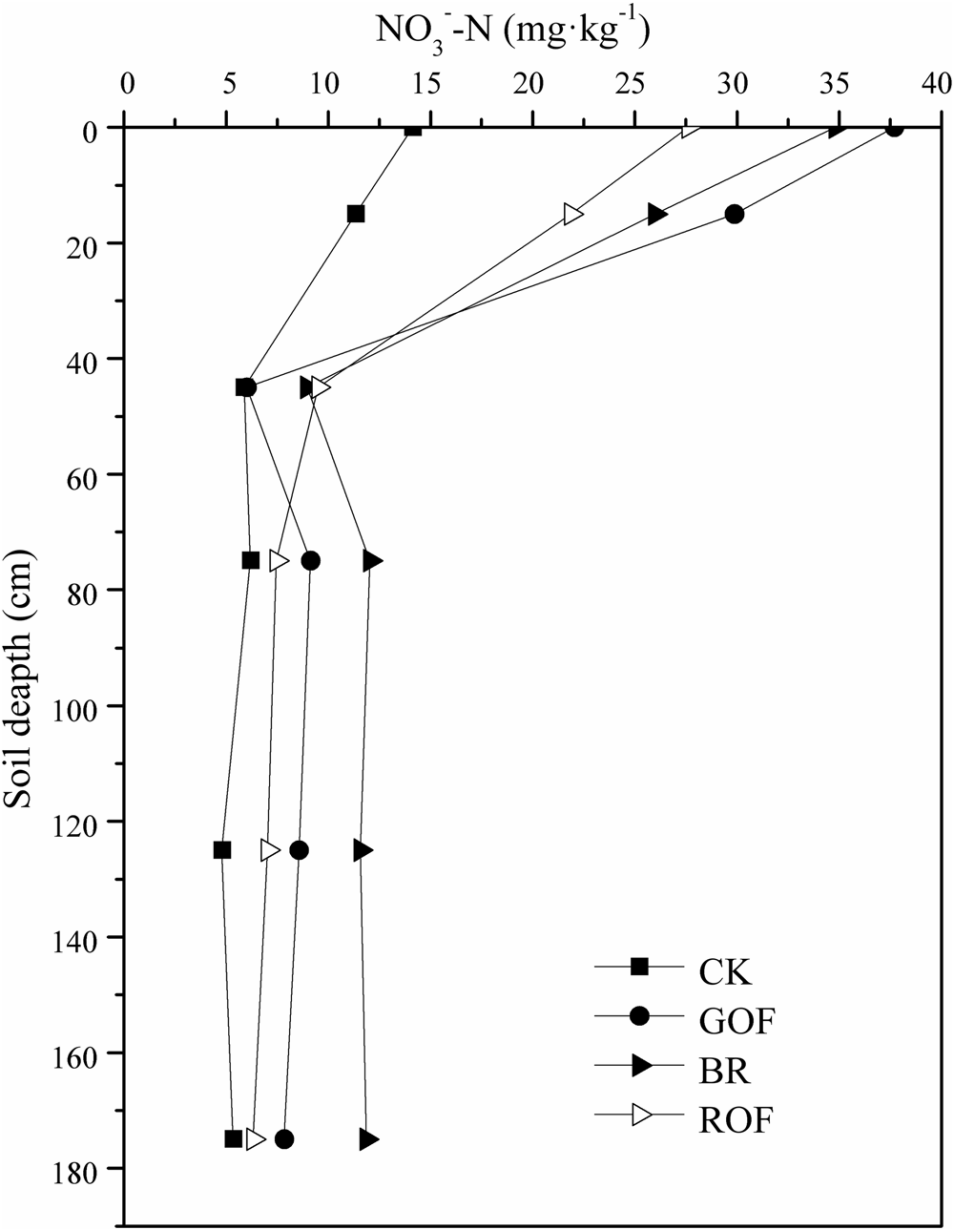
Nitrate content in soil profile after harvest the third rotation (with depth).

Organic fertilizer significantly affected soil NO_3_^−^-N concentration in the 0–175 cm soil layers (Fig. 5). Due to incomplete utilization of fertilizer, treatments with the organic fertilizers increased the nitrate content in soil profile especially in the top layers compared with CK. Topsoil had the most obvious effect, above all GOF treatment reached 29.9 mg·kg^−1^, and this may be result from nitrification and mineralization for its instability^20^. Conversely, owing to the higher humification and stability degree of ROF^31^, nitrate content in ROF treatment was lower than any other fertilization treatments. After application of BR and GOF, nitrate had a dramatic enrichment in deep soil (especially below 75 cm) indicating N surplus, and such accumulation of NO_3_^−^-N in soil profile posed a high risk of N leaching into groundwater. BR and GOF were all incomplete fermentation without thermophilic phase, and then they had a low humification degree with very little stabilized organic matters^32^. Thus a large amount of nitrogen in BR and ROF cannot be fixed like ROF, which lead to nitrate leaching seriously. In ROF treatment, nitrate content in the soil below 100 cm almost had no difference with CK. From the perspective of security, ROF is the environmentally friendly way for fertilizing.

### Nitrogen balance and N translocation

Calculation of N balance is one potentially useful method for predicting the risk of nitrate leaching into groundwater^10^. N balance in each treatment was calculated under this tomato-celery rotation system (Table 2). Without fertilizer, the amount of N_min_ could achieve about 145 kg·ha^−1^. However, the N_residual_ level was lower than the N_initial_ level, indicating soil N depletion in some degree. N_uptake_ in the fertilization treatments were higher than that in CK treatment, especially in the ROF treatment, showing that fertilization can promote the absorption of N by root. After organic fertilizer application, the residual NO_3_^−^-N in the 0–60 cm soil layer after crop harvest accumulated to 100–122 kg·ha^−1^. Although this was still higher than the environmental safety standard in Europe (90–100 kg·ha^−1^ in the 0–100 cm soil layer)^10^, it resulted in 50–75% less NO_3_^−^-N accumulation in the soil profiles than the initial soil, and therefore the environmental risk was reduced in some degree. These results suggested that organic fertilizer application could be benefit for crop uptake, reduce the NO_3_^−^-N in the soil and then alleviate the soil NO_3_^−^-N leaching. NUE in these fertilization treatments were 19.4–30.0%, the ROF treatment presented the highest NUE among all the treatments due to the highest uptake by crops, implying the optimum fertilization way.

**Table 2:** N balance and N translocation from soil and fertilizer to vegetable.

Treatment	N_initial_	N_input_	N_min_	N_uptake_	N_residual_	N_utilization_	NUE
	(kg·ha^−1^)	(kg·ha^−1^)	(kg·ha^−1^)	(kg·ha^−1^)	(kg·ha^−1^)	(kg·ha^−1^)	(%)
CK	396 ± 18	0	145 ± 12	481 ± 21	60 ± 10	—	—
GOF		1050		593 ± 34	122 ± 10	204	19.4
BR		1050		600 ± 26	120 ± 18	208	19.8
ROF		1050		726 ± 28	100 ± 15	314	30.0

Values are given as the mean ± standard deviation (n = 9).

### Nitrate concentration of soil leachate

Although the organic fertilizer application could be benefit for crop uptake and reduce the NO_3_^−^-N in the soil, soil are still at the high risk of leaching with the high N_residual_ and low NUE in all the fertilization treatments. Then the NO_3_^−^-N concentration of soil leachate at 100 cm depth below the soil surface were detected after harvest vegetables. NO_3_^−^-N concentration of soil leachate varied with treatments and crop types, ranging from 6.3 to 35.1 mg·L^−1^ for tomato and from 4.2 to 30.3 mg·L^−1^ for celery (Table 3). Soil NO_3_^−^-N leaching in tomato seasons was generally higher than in celery seasons due to higher crop N uptake and higher evaporation in celery rotation leading to less drainage into deeper layers. NO_3_^−^-N leaching in all treatments decreased after application of organic fertilizers especially ROF. Fertilizer type significantly affects the NO_3_^−^-N concentration in the soil leachate. The least NO_3_^−^-N leaching was observed in the ROF treatment mainly due to ROF with a higher organic matter content and biological activity, stabilization and humification degree, resulting in the increase of soil aggregation, nitrogen fixation capacity and decrease of NO_3_^−^-N leaching^25^. Moreover, NO_3_^−^-N concentration in CK and ROF treatments dropped below 10 mg·L^−1^ after harvest the second rotation (celery), which satisfied the threshold (<10 mg·L^−1^) for drinking water set by the US Environmental Protection Agency. This result suggested that application of ROF was no more likely to impair groundwater quality than the GOF, BR or even CK treatments.

**Table 3:** NO_3_^−^-N concentrations in the soil leachate after harvest the vegetables (mg·L^−1^).

Treatment	Original	Tomato 1	Celery	Tomato 2
CK	38.1 ± 0.2	10.2 ± 0.2	4.2 ± 0.1	6.3 ± 0.1
GOF	38.2 ± 0.1	33.8 ± 0.3	28.5 ± 0.2	31.6 ± 0.3
BR	37.2 ± 0.3	35.1 ± 0.4	30.3 ± 0.3	32.1 ± 0.3
ROF	37.4 ± 0.3	11.6 ± 0.2	8.2 ± 0.1	6.4 ± 0.2

Values are given as the mean ± standard deviation (n = 9).

## Conclusions

Organic fertilizers significantly increased vegetable yield and quality, but with inappropriate application may cause serious environmental risk. Maximum vegetable yield of 80.9, 68.3, 112.7 t·ha^−1^ (first, second and third rotation crop, respectively) with best vegetable quality was obtained in ROF treatment. The highest N use efficiency with the least nitrate enrichment in soil was also found in ROF treatment. Moreover, under this fertilization way, nitrate concentration in soil leachate satisfied the threshold for drinking water. Thus, ROF was suggested to be the optimal fertilizer with the best yield, quality and the least environmental risk under the “tomato-celery” rotation system.

## Materials and methods

### Site description

One and a half years of field experiments (tomato1-celery-tomato2) were conducted on clay loam soil at Liuminying Agricultural Ecological Station (39°41′ N, 116°34′ E) in southeast suburb area (Daxing district) of Beijing, northwest edge of North China Plain. The soil was calcareous, alkaline, and rich in phosphorus and potassium. Agriculture in the area is intensified by a double cropping system (two vegetables a year) with high-yielding cultivar and high inorganic fertilizer (more than 1000 kgN·ha^−1^·yr^−1^) input. Some of the characteristics of this soil were determined before this experiment (Table 1). The average air temperature during tomato planting period was about 25 °C, while in celery planting period was about 18 °C.

### Crop rotation and experimental fertilizers

A typical spring tomato–autumn celery double cropping rotation was chosen, representative of the common farming practices in the area, where tomato is usually planted from March to July and celery from August to October. Tomato cultivar with Israel 1420 greenhouse grown tomato (Lycopersicon esculentum Mill.) was planted in the experimental plot (see Section 2.3) at a density of 36,000 plant·ha^−1^. After tomato harvest, soil was ploughed before planting autumn celery. Celery cultivar with California celery (Apium graveolens L) was planted in the experimental plot at a rate of 2,300,000 plant·ha^−1^. The selected crop varieties and planting densities is representative of that used by local farmers.

In order to evaluate of agronomic and ecological effects of soil amendment, three kinds of common organic material i.e. general organic fertilizer (GOF), biogas residue (BR) and refined organic fertilizer (ROF) were used as N fertilizer. GOF was made by chicken manure and corn stalk through water-logged composting; BR was taken from Liuminying Biogas Station, which was made by chicken manure through anaerobic digestion; and ROF was made by chicken manure and mushroom residue through a 90 days thermophilic aerobic composting. Some of the composition and characteristics of these organic fertilizers are given in Table 4.

**Table 4:** Composition and characteristics of the three organic fertilizers.

Fertilizer	GOF	BR	ROF
Organic matter (g·kg^−1^)^a^	356 ± 3	318 ± 2	404 ± 5
Humic acid (g·kg^−1^)^a^	53.9 ± 1.7	61.4 ± 1.8	145 ± 7
Fulvic acid (g·kg^−1^)^a^	72.1 ± 2.5	59.7 ± 1.5	29.8 ± 0.9
Total N (g·kg^−1^)^a^	12.2 ± 0.2	13.6 ± 0.3	20.1 ± 0.2
Total P (g·kg^−1^)^a^	23.1 ± 0.3	51.0 ± 0.4	23.6 ± 0.2
Total K (g·kg^−1^)^a^	13.7 ± 0.2	9.0 ± 0.1	20.2 ± 0.2
Total Cu (mg·kg^−1^)^a^	128 ± 1.7	89.1 ± 1.2	75.7 ± 0.9
Total Zn (mg·kg^−1^)^a^	210 ± 1.4	248 ± 0.7	181 ± 1.4
Total As (mg·kg^−1^)^a^	4.12 ± 0.19	3.28 ± 0.29	7.85 ± 0.47
Total Cd (mg·kg^−1^)^a^	2.04 ± 0.42	2.33 ± 0.07	2.15 ± 0.11
Germination index (%)	83.5 ± 3.4	97.2 ± 2.8	112 ± 4.3
Escherichia coli (logCFU·g^−1^)	3.12 ± 0.15	2.33 ± 0.17	ND
Moisture content (%)	43.8 ± 0.2	45.2 ± 0.1	35.2 ± 0.2

Values are given as the mean ± standard deviation (n = 3). ^a^Based on dry matter (DM). ND: not detected.

### Experimental design

The experiment was conducted in a vegetable greenhouse during the tomato and celery growing season. Four treatments with three replicates were carried out, namely CK, GOF, BR and ROF. Then the experimental area consisted of 12 plots, 5.5 m wide and 6 m long for each, and these 12 plots were arranged as split plots in a randomized complete block with a 0.5 m isolation strip in order to avoid interference. The CK was a control treatment without fertilization. GOF, BR and ROF treatments were applied with the same amount of N with 350 kgN·ha^−1^ for each crops. Previous study has found that top dressing can increase the crop yield^20^. Then in this experiment, 66.7% of the fertilizer was used as base fertilizer and the remaining 33.3% as top dressing in fruit swelling period and vigorous period for tomato and celery, respectively. The management practices for controlling pest, disease and weeds complied with local practices for high-yield production.

### Analytical methods

Tomato and celery plant were sampled from a 5 m^2^ area in each plot at harvest for the measurements of vegetable (tomato and celery) yield and tomato residual biomass. Samples of vegetable and tomato residual were oven-dried at 65 °C until they reached a constant weight to determine the water content and dry matter. The N content in vegetable and tomato residual of the samples were determined by the micro-Kjeldahl method by digesting the sample in H_2_SO_4_-H_2_O_2_ solution^33^. N uptake by plants was estimated by multiplying the tomato, tomato residual and celery dry matter weight by their N concentrations.

Three tomatoes (or three plants of celery) per plot with similar degree of maturity and similar size and without external defects were picked for the quality indices (mainly taste quality, nutrient quality and safety quality) measurement. Tomatoes or celeries were squeezed in a blender, and then the content of vitamin C (Vc), soluble sugar (SE), and titratable acidity (TA) in the plants were detected according to^34^. Besides, some of the squeezed vegetable was extracted with deionized water, filtered and then the concentration of NO_3_^−^-N in vegetable was determined by a continuous-flow analyzer (TRAACS 2000, Bran and Luebbe, Norderstedt, Germany).

For soil N measurements, three ceramic candle extraction systems with tubes (inside diameter 50 mm) were installed in each plot at 100 cm soil depths. The amount of nitrate leached during the growing season may be minimal compared to leaching losses that occur between the harvest of one crop and the planting of the next^23^. Then samples of the soil leachate were taken after each harvest and/or before sowing. Furthermore, soil samples in all plots were taken after each harvest and/or before each planting by sampling three cores per plot with an auger (3 cm inside diameter tube) to 60 cm depth in 30 cm increments. Moreover, soil samples in the depth of 15, 45, 75, 125 and 175 cm were taken after harvesting the second batch of tomato (tomato 2) to research the change of nitrate with soil depth. Soil samples obtained from the same layer and plot were thoroughly mixed. All of the soil and soil leachate samples were immediately brought to the laboratory for the measurement of NO_3_^−^-N and soil moisture content.

Each fresh soil sample was extracted with CaCl_2_^35^, and the concentration of nitrate was determined by a continuous-flow analyzer (TRAACS 2000, Bran and Luebbe, Norderstedt, Germany). Soil samples were dried to a constant weight in an oven at 105 °C to determine the water content and dry matter. Bulk density of the soils was measured in the 0–60 cm soil depth with soil cores (3 cm inside diameter by 20 cm long). The NO_3_^−^-N contents in soil (mg·kg^−1^) were converted to kg·ha^−1^ based on the bulk density of different soil layers in order to calculate the N balance. For the nitrate analysis of soil leachate, the water samples were filtered through 0.45 μm membranes and the concentration of nitrate was determined by a continuous-flow analyzer^36^.

### Nitrogen balance

Items in the N balance were estimated in each plot during the whole crop growing seasons. NO_3_^−^-N below 60 cm soil depth and NH_4_^+^-N throughout the soil profile will not be included in the N balance calculations because the crop roots in this experiment were mainly distributed in the 0–60 cm depth and relatively low changes in NH_4_^+^-N content between seasons were found (data not presented). The N balance can be written as:

1 $${{\rm{N}}}_{{\rm{initial}}}+{{\rm{N}}}_{{\rm{input}}}+{{\rm{N}}}_{{\rm{\min }}}-{{\rm{N}}}_{{\rm{uptake}}}-{{\rm{N}}}_{{\rm{residual}}}={{\rm{N}}}_{{\rm{surplus}}}$$

where N_initial_ is initial soil NO_3_^−^-N in the 0–60 cm soil profiles; N_input_ is N application rate (350 kg N·ha^−1^ per rotation crop plus 3 rotation crops); N_min_ is N mineralization; N_uptake_ is N uptake by plant; N_residual_ is residual NO_3_^−^-N in 0–60 cm soil profiles, and N_surplus_ represent N that store in various soil fraction (mainly organic N) and N loss. N loss is considered as mainly NO_3_^−^-N leaching, since other N losses via denitrification, volatilization and erosion are relatively low under such environmental conditions as reported by Fang *et al*.^37^.

N mineralization (N_min_) was estimated by the balance of N inputs and outputs in the control (CK) as follows:

2 $${{\rm{N}}}_{{\rm{\min }}}={{\rm{N}}}_{{\rm{uptake}},0}+{{\rm{N}}}_{{\rm{residual}},0}-{{\rm{N}}}_{{\rm{initial}},0}$$

where N_uptake,0_, N_residual,0_ and N _initial,0_ are crop N uptake, residual and initial soil NO_3_^−^-N in the 0–60 cm soil profile of the control, respectively.

3 $${{\rm{N}}}_{{\rm{utlization}}}={{\rm{N}}}_{{\rm{input}}}-{{\rm{N}}}_{{\rm{surplus}}}$$

4 $${\rm{NUE}}={{\rm{N}}}_{{\rm{utlization}}}/{{\rm{N}}}_{{\rm{input}}}$$

N_utlization_ is the part of N_uptake_ offered by organic fertilizer. NUE is the fertilizer N use efficiency during the one and a half years of experiment period.

### Statistical analyses

Analysis of variance (ANOVA) was performed with the SAS8.2 for Windows, and mean comparisons were done using the least significant difference (LSD) test at P < 0.05.

### Data availability statement

The authors declared that none of the data in the paper had been published or was under consideration for publication elsewhere.
